# Emotion dysregulation in youths with obsessive-compulsive disorder and its implication for treatment - An exploratory study from the TECTO trial: A protocol and statistical analysis plan

**DOI:** 10.1016/j.conctc.2024.101408

**Published:** 2024-12-04

**Authors:** Christine Lykke Thoustrup, Camilla Uhre, Valdemar Uhre, Melanie Ritter, Signe Vangkilde, Janus Engstrøm, Jane Lindschou, Christian Gluud, Anne Katrine Pagsberg, Markus Harboe Olsen

**Affiliations:** a Child and Adolescent Mental Health Center, Copenhagen University Hospital, Mental Health Services CPH, Gentofte Hospitalsvej 3A, 1. Sal, 2900, Hellerup, Denmark; b Department of Clinical Medicine, Faculty of Health and Medical Sciences, University of Copenhagen, Blegdamsvej 3B, 2200, Copenhagen, Denmark; c Center for Clinical Neuropsychology, Children and Adolescents, Copenhagen University Hospital, Rigshospitalet, Blegdamsvej 9, 2100, Copenhagen, Denmark; d Danish Research Centre for Magnetic Resonance, Centre for Functional and Diagnostic Imaging and Research, Copenhagen University Hospital - Amager and Hvidovre, Kettegård Allé 30, Afsnit 714, 2650, Hvidovre, Denmark; e Department of Psychology, University of Copenhagen, Center for Sundhed og Samfund, Øster Farimagsgade 2A, 1353, Copenhagen, Denmark; f Copenhagen Trial Unit, Centre for Clinical Intervention Research, The Capital Region, Copenhagen University Hospital, Rigshospitalet, Copenhagen, Denmark; g Department of Regional Health Research, The Faculty of Health Sciences, University of Southern Denmark, Odense, Denmark; h Department of Neuroanaesthesiology, The Neuroscience Centre, Copenhagen University Hospital, Rigshospitalet, Copenhagen, Denmark

## Abstract

**Background:**

Research on improving psychotherapy for youths with obsessive-compulsive disorder (OCD), including cognitive behavioral therapy (CBT), should explore what works for whom and how by examining baseline moderators and potential mechanisms of change. Emotion dysregulation is proposed as an intermediate therapy factor in a transdiagnostic framework. This study investigates emotion dysregulation as an outcome, mechanism, and moderator of psychotherapy in youths aged 8–17 years with OCD.

**Methods:**

Data are from a randomized clinical trial and a parallel prospective study of healthy controls. Participants with OCD (n = 130; 121 in this study) were randomized to 14 sessions of either family-based CBT with exposure and response prevention versus family-based psychoeducation and relaxation training. We will; 1) assess if emotion dysregulation, measured by the Difficulties in Emotion Regulation Scale (DERS), decreases from baseline to end-of-treatment; 2) compare the proportion of participants with normative emotion regulation to a 90% reference interval from healthy controls (n = 90); 3) use linear regression to examine if baseline emotion dysregulation moderates treatment effects measured by the Children's Yale-Brown Obsessive-Compulsive Scale; 4) investigate if changes in emotion dysregulation mediate treatment effects; and 5) investigate the stability of emotion regulation over time in the healthy controls. Analyses 1–4 will be conducted for all OCD participants and separately for the two treatment groups. Two independent investigators will perform the analyses.

**Conclusion:**

This protocol and statistical analysis plan are presented to enhance analytical transparency and limit bias.

## 1 Introduction

Obsessive-compulsive disorder (OCD) is a common psychiatric condition affecting up to 3% of youths with significant impact across several life domains [1]. Psychological interventions have proven effective in alleviating OCD symptoms with cognitive behavioral therapy (CBT) with exposure and response prevention as the recommended first-line treatment [2]. However, up to 40% of youths with OCD do not or only partially respond to treatment [3]. To improve treatment effect, clinical research should investigate whether certain factors affect the mechanisms of change in psychotherapy and whether some youths are more likely to benefit from such treatments. In addition to obsessive thoughts and compulsive behaviors, findings from cross-sectional studies suggest that youths with OCD also experience difficulties in regulating their emotions – i.e., emotion dysregulation [4,5]. Emotion dysregulation encompass difficulties in differentiating and accepting the full range of one's emotions and emotional responses and difficulties controlling maladaptive impulses or behaviors when being upset [6]. Thus, investigating emotion dysregulation could be of interest when exploring factors affecting treatment outcome. To date, the limited research within this area yields conflicting results. Two studies suggest that pre-treatment emotion dysregulation in youths (aged 7–17 years) may negatively impact treatment response [7,8] while others suggest no such impact [9,10]. Besides investigations of the predictive and moderating effects of emotion regulation, no study has explored whether change in emotion dysregulation during treatment is related to successful treatment outcome for youths with OCD. In the present study, emotion dysregulation will be assessed by the Difficulties in Emotion Regulation Scale (DERS) [6], a commonly used questionnaire for assessing emotion dysregulation in youths with mental health problems [11,12]. The DERS has previously been used in both adult and pediatric OCD cohorts, demonstrating good psychometric properties and moderate to strong associations with obsessive-compulsive symptomatology [5,[13], [14], [15], [16]].

Here, we describe the protocol and statistical analysis plan for the exploratory study from the TECTO trial focusing on emotion dysregulation in youths with OCD [17]. In the present study we will investigate if (1) emotion dysregulation in participants is ameliorated from baseline to end-of-treatment; (2) the proportion of participants reporting normative levels of emotion regulation increases from baseline to end-of-treatment; (3) pre-treatment levels of emotion dysregulation moderate treatment outcome and progress; (4) the effect of psychotherapy on OCD outcome is mediated through changes in emotion dysregulation; and (5) we will investigate the natural change of emotion regulation abilities in healthy controls not receiving psychotherapy. For analyses 1 to 4, each analysis will first be investigated by merging all TECTO participants into one cohort and second by investigating each treatment group separately comparing Family-based cognitive behavioral therapy (FCBT) versus family-based psychoeducation and relaxation training (FPRT).

## 2 Methods

### 2.1 Study design

Data in this study come from our randomized clinical trial (RCT) including 130 patients with OCD (of which 121 participated in this study due to later onset of data collection) and a parallel prospective observational study including 90 healthy controls aged 8–17 years [17]. In the RCT, participants were randomized stratified by age (8–12 and 13–17 years) and baseline Children's Yale-Brown Obsessive-Compulsive Scale (CY-BOCS) [18] total score at baseline (16–23 and ≥24 points). The trial was registered on ClinicalTrials.gov (identification no. NCT03595098; 23 July 2018) before the inclusion of the first participant. For further details, please see our published protocol and statistical analysis plan [17,19]. In accordance with the guidelines for the DERS, only adolescents aged 11 and older were included in this study.

### 2.2 TECTO trial participants

All participants eligible for trial inclusion had (1) a primary diagnosis of OCD (ICD-10 F42) (confirmed by a diagnostic assessment using the Kiddie-Schedule for Affective Disorders and Schizophrenia – Present and Lifetime Version, K-SADS-PL, [20]) and (2) at least moderate clinical severity (entry score ≥16 on the CY-BOCS). Patients were excluded if they had comorbid pervasive developmental disorder not including Asperger's syndrome, schizophrenia/paranoid psychosis, mania or bipolar disorder, depressive psychotic disorders, or substance dependence syndrome. Additional exclusion criteria were IQ < 70, treatment with CBT, psychoeducation with relaxation training, serotonin reuptake inhibitors, or other antidepressant or antipsychotic medication within the last six months prior to trial entry.

### 2.3 Trial interventions

Participants were randomized to receive 14 sessions of 75 min each of FCBT versus FPRT. The key components of the manualized FCBT are exposure and response prevention, family involvement, psychoeducation, and homework assignments. FPRT is the active control intervention consisting of relaxation training, family involvement, psychoeducation, and home assignments [21]. Hence, the intended difference between the two interventions in the TECTO trial is the absence of the exposure and response prevention element in the FPRT group, which is suggested as the key treatment component of the FCBT group [17]. Both treatment manuals are described in detail in our published trial protocol [17]. In this study, the participants will first be investigated as one cohort of youths receiving psychotherapy, and second assessed for each treatment group separately.

### 2.4 Healthy controls

Healthy controls were recruited from the regional population via a random age-matched sample of children and adolescents, drawn from the Danish Central Person Registry [22]. They were matched 1:1 on age and sex to participants with OCD. Exclusion criteria were (1) any current or lifetime psychiatric diagnoses verified with K-SADS-PL or (2) meeting any exclusion criteria used for participants with OCD.

### 2.5 Outcomes

#### 2.5.1 Emotion dysregulation

Emotion dysregulation is assessed using self-reports of the Difficulties in Emotion Regulation Scale (DERS) at baseline and end-of-treatment [6]. The DERS comprises 36 items indexing six dimensions of emotion dysregulation. Each item is rated on a five-point Likert scale, which sum up to an overall total score (DERS-T) ranging from 36 to 180, with higher scores indicating more emotion dysregulation. The DERS has shown good test-retest reliability over 6–8 weeks in college students and adolescents, and has been validated with children down to the age of 11 years [6,23]. Self-report versions show good internal consistency (Cronbach's α = .84) [23]. Following the guidelines from the original study by Gratz and Roemer [6] we will present results from the DERS-T using summed scores in the main analyses, which will enable us to make the most accurate comparisons with other studies. However, we recognize that a subset of previous literature suggests removing the subscale reflecting the Awareness dimension due to weak psychometric properties thereby creating a revised total score (DERS-T Revised) of the remaining five subscales [24]. Thus, to enhance comparability, generalizability, and provide data for future synthetization of results, we will include analyses with the DERS-T Revised and present all six subscale scores in supplementary material.

#### 2.5.2 Normative levels of emotion regulation

Normative levels of emotion regulation are estimated from the 90% reference interval for DERS-T for the healthy control group at end of study. The 90% reference interval is calculated by multiplying the standard deviation of the sample by a *z* of 1.645. Since higher scores on the DERS reflect more emotion dysregulation, we will only estimate the upper reference level. Thus, 5% of all results falling above the reported reference interval will be flagged as “abnormal” or in this case as emotionally dysregulated. The remaining 95% of all results from the healthy populations will be flagged as normative levels of emotion regulation.

#### 2.5.3 Treatment outcome

Treatment outcome is defined as severity of OCD symptoms at end-of-treatment and as change in OCD symptoms from baseline to end-of-treatment (see *Statistical models* for details). OCD symptoms are assessed at baseline and end-of-treatment using the clinician-rated, semi-structured interview CY-BOCS [18]. CY-BOCS captures OCD symptoms during the past week by assessing frequency, interference, distress, resistance, and control on a five-point Likert scale across two subscales for obsessions and compulsions, respectively. The subscales are combined to create a total score of OCD severity, ranging from 0 to 40 with higher scores indicating higher symptom severity. The CY-BOCS has demonstrated high internal consistency, with total score alphas ranging from 0.87 to 0.90 [18,25]. The CY-BOCS has also been shown to be treatment sensitive [26].

#### 2.5.4 Stability of emotion regulation

Stability of emotion regulation over time will be assessed as the mean difference between baseline and end-of-study in DERS-T for the healthy controls.

### 2.6 Statistical models

The statistical models are summarized in Table 1, and presented using DERS-T, while sensitivity analyses will be carried out using DERS-T revised.

**Table 1:** Statistical models.

Change in emotion dysregulation after treatment	
Model 1a - all participants	Paired *t*-test comparing the mean difference between DERS-T at baseline and end-of-treatment
	H_0_: DERS-T at baseline = DERS-T at end-of-treatment
	H_1_: DERS-T at baseline ≠ DERS-T at end-of-treatment
Model 1b - by allocation	Linear regression model
	*Outcome:* DERS-T end-of-treatment
	*Fixed effects:* Allocation (0 = FPRT, 1 = FCBT), DERS-T at baseline; and CY-BOCS (16–23 compared to ≥ 24 points) at baseline
**Normative levels of emotion regulation after treatment**	
Model 2a – all participants	McNemar mid-*p*
	H_0_: Proportion of ED_pre_ = Proportion of ED_post_
	H_1_: Proportion of ED_pre_ ≠ Proportion of ED_post_
Model 2b – by allocation	Logistic regression model
	*Outcome:* Within or outside the reference interval from Study Norms
	*Fixed effects:* Allocation (0 = FPRT, 1 = FCBT), DERS-T at baseline, CY-BOCS (16–23 compared to ≥ 24 points) at baseline
**The moderating effect of pre-treatment emotion dysregulation on treatment outcome and symptom change**	
Model 3a – all participants	Linear regression model
	*Outcome:* CY-BOCS at end-of-treatment
	*Fixed effects:* DERS-T at baseline, CY-BOCS at baseline
	*Interaction link:* CY-BOCS at baseline and DERS-T at baseline
Model 3c – by allocation	Linear regression model
	*Outcome:* CY-BOCS at end-of-treatment
	*Fixed effects:* Allocation (0 = FPRT, 1 = FCBT), DERS-T at baseline, CY-BOCS at baseline
	*Interaction link:* Allocation and DERS-T at baseline
Model 3b - all participants	Mixed effects linear regression model
	*Outcome:* CY-BOCS (week 0, 4, 8, 16)
	*Fixed effects:* Time (0, 4, 8, and 16 weeks), DERS-T at baseline
	*Random effects* (slope and intercept): Participant
	*Interaction link:* Time and DERS-T at baseline
Model 3d – by allocation	Mixed effects linear regression model
	*Outcome:* CY-BOCS (week 0, 4, 8, 16)
	*Fixed effects:* Allocation (0 = FPRT, 1 = FCBT), time (0, 4, 8, and 16 weeks), DERS-T at baseline
	*Random effects* (slope and intercept): Participant
	*Interaction link:* Allocation and DERS-T at baseline
**The mediating effect of changes in emotion dysregulation on treatment outcome**	
Model 4a – all participants	*Step 1 - path c*
	*Outcome:* CY-BOCS at end-of-treatment
	*Fixed effects*: CY-BOCS at baseline
	*Step 2 – path a*
	*Outcome:* DERS change (T2-T1)
	*Fixed effects:* CY-BOCS at baseline
	*Step 3 – path b*
	*Outcome:* CY-BOCS end-of-treatment
	*Fixed effects:* DERS change (T2-T1), CY-BOCS at baseline
	*Step 4 – path c’*
	*Outcome:* CY-BOCS at end-of-treatment
	*Fixed effects*: CY-BOCS at baseline, DERS change (T2-T1)
Model 4b – by allocation	*Step 1 - path c*
	*Outcome:* CY-BOCS at end-of-treatment
	*Fixed effects*: Allocation (0 = FPRT, 1 = FCBT), CY-BOCS at baseline
	*Step 2 – path a*
	*Outcome:* DERS change (T2-T1)
	*Fixed effects: A*llocation (0 = FPRT, 1 = FCBT)
	*Step 3 – path b*
	*Outcome:* CY-BOCS end-of-treatment
	*Fixed effects:* DERS change (T2-T1), Allocation (0 = FPRT, 1 = FCBT)
	*Step 4 – path c’*
	*Outcome:* CY-BOCS at end-of-treatment
	*Fixed effects*: Allocation (0 = FPRT, 1 = FCBT), DERS change (T2-T1)
**Stability of emotion regulation over time in healthy controls**	
Model 5	Paired *t*-test comparing the mean difference between DERS-T at baseline and end-of-study for healthy controls
	H_0_: DERS-T at baseline = DERS-T at end-of-study
	H_1_: DERS-T at baseline ≠ DERS-T at end-of-study

#### 2.6.1 Change in emotion dysregulation after treatment

First, we will investigate if emotion dysregulation decreases from baseline to end-of-treatment in all participants by running a paired *t*-test using DERS-T at baseline and end-of-treatment (Table 1; *model 1a*). Second, to ascertain if there is a significant difference between the treatment groups (FCBT versus FPRT) in DERS-T at end-of-treatment, we will use a linear regression model with DERS-T at end-of-treatment as outcome; and allocation, DERS-T at baseline, and CY-BOCS (16–23 compared to ≤ 24 points) at baseline as fixed effects (Table 1; *model 1b*).

#### 2.6.2 Normative levels of emotion regulation after treatment

To determine if the proportion of participants with OCD reporting normative levels of emotion regulation increases from baseline to end-of-treatment, we will label the participants as being within or outside the reference interval. We will use the McNemar mid-*p* test to investigate if the proportion of participants within the reference interval is higher after the intervention compared to baseline (Table 1; *model 2a*). To ascertain if there is a difference in normative proportion change between the treatment groups, we will use a logistic regression model with within or outside the reference interval as the outcome; and allocation, DERS-T at baseline, and CY-BOCS at baseline as fixed effects (Table 1; *model 2b*).

#### 2.6.3 The moderating effect of pre-treatment emotion dysregulation on treatment outcome and symptom change

To determine if pre-treatment levels of emotion dysregulation moderate the effect of psychotherapy measured by OCD symptom severity at end-of-treatment, we will conduct a linear regression model with CY-BOCS at end-of-treatment as the outcome; DERS-T at baseline, and CY-BOCS at baseline as fixed effects; and an interaction link between CY-BOCS at baseline and DERS-T at baseline. If the interaction between CY-BOCS at baseline and DERS-T at baseline is significant, then pre-treatment levels of emotion dysregulation are deemed a moderator of CY-BOCS week 16 adjusted for baseline CY-BOCS (Table 1; *model 3a*). Furthermore, to determine if pre-treatment levels of emotion dysregulation moderate symptom change during psychotherapy, we will conduct a mixed effects linear regression model with all CY-BOCS values (week 0, 4, 8, 16) as the outcome; time, and DERS-T at baseline as fixed effects; participants as random effects; and an interaction link between time and DERS-T at baseline. If the interaction between time and DERS-T at baseline is significant, then pre-treatment levels of emotion dysregulation is deemed a moderator of the change of CY-BOCS over time (Table 1; *model 3b*).

Moreover, to ascertain if there is a difference between the treatment groups in the moderating effects of DERS-T we will use a linear regression model with CY-BOCS at end-of-treatment as the outcome; and allocation, and DERS-T at baseline as fixed effects; and an interaction link between allocation and DERS-T at baseline. If the interaction link between allocation and DERS-T at baseline is significant, DERS-T at baseline influences CY-BOCS at end-of-treatment differently for each group (Table 1; *model 3c*). Finally, to ascertain if there is a difference between the treatment groups, we will use a mixed effects linear regression model with all CY-BOCS values as the outcome; and allocation, time, and DERS-T at baseline as fixed effects; participants as random effects; and an interaction links between allocation and DERS-T at baseline. If the interaction link between allocation and DERS-T at baseline is significant, DERS-T at baseline influences the symptom change in the treatment groups differently (Table 1; *model 3d*).

#### 2.6.4 The mediating effect of changes in emotion dysregulation on treatment outcome

To determine if the effect of psychotherapy is mediated by changes in emotion dysregulation during therapy, we will conduct a mediation analysis. The mediator investigated is the difference between DERS-T at end-of-treatment and DERS-T at baseline. The outcome is CY-BOCS at end-of-treatment; and CY-BOCS at baseline is a fixed effect. We will conduct the mediation analysis by following the original 4-steps methods suggested by Baron and Kenny [27] (Table 1, *model 4a*). *Step 1*, we estimate the relationship between psychotherapy and CY-BOCS at end-of-treatment with CY-BOCS at baseline as a fixed effect (Table 1, *model 4a, path c*). The coefficient of *path c* must be significantly different from 0, indicating that CY-BOCS at end-of-treatment has changed from baseline, for the mediation analysis to proceed. *Step 2*, we estimate the relationship between baseline CY-BOCS and change in emotion dysregulation (Table 1, *model 4a, path a*). The coefficient for *path a* must be significantly different from 0, indicating that psychotherapy predicts emotion dysregulation change, for the mediation analysis to proceed. *Step 3*, we estimate the relationship between change in emotion dysregulation and CY-BOCS at end-of-treatment while controlling for CY-BOCS at baseline (Table 1, *model 4a, path b*). The coefficient of *path b* must be significantly different from 0, indicating that change in emotion dysregulation predicts CY-BOCS at end-of-treatment while controlling for CY-BOCS at baseline, for the mediation analysis to proceed. *Step 4*, we estimate the relationship between CY-BOCS at baseline and CY-BOCS at end-of-treatment while controlling for change in emotion dysregulation (Table 1, *model 4a, path c’*). The coefficient of *path c’* should be smaller than the coefficient in *path c* and ideally non-significant and near 0 for change in emotion dysregulation to be deemed a mediator. If the effect of the mediator is significant indicated by the regression coefficient for path c’ being smaller than the regression coefficient for path c, we will assess its effect by multiplying regression coefficients from path a and path b and estimate the 95 % bootstrap confidence intervals and the effect size expressed as kappa squared as proposed by Preacher and Kelly [28].

Furthermore, to ascertain if there is a difference between the treatment groups, we will conduct a new mediation analysis with CY-BOCS at end-of-treatment as outcome; allocation as fixed effect, and emotion dysregulation change as a potential mediator (Table 1, *model 4b*). *Step 1*, we estimate the relationship between allocation and CY-BOCS at end-of-treatment (Table 1, *model 4b, path c*). *Step 2*, we estimate the relationship between allocation and emotion dysregulation change (Table 1, *model 4b, path a*). *Step 3*, we estimate the relationship between emotion dysregulation change and CY-BOCS at end-of-treatment while controlling for allocation (Table 1, *model 4b, path b*). Step 4, we estimate the relationship between allocation and CY-BOCS at end-of-treatment while controlling for emotion dysregulation change (Table 1, *model 4b, path c’*). If the effect of the mediator is significant indicated by the regression coefficient for path c’ being smaller than the regression coefficient for path c, we will assess its effect by multiplying regression coefficients from path a and path b and estimate the 95 % bootstrap confidence intervals and the effect size expressed as kappa squared (Table 1; *model 4b*).

#### 2.6.5 Stability of emotion regulation over time in healthy controls

To investigate the stability of emotion regulation in this age group, we will carry out a paired *t*-test comparing DERS-T for healthy controls at baseline and end-of-study (Table 1; *model 5b*) and present the mean difference together with 95% confidence intervals. Furthermore, we will present the absolute difference together with 95% confidence intervals. Finally, for simplicity we will calculate reliability using intraclass correlation coefficient as a simplified measure of reliability [29]. Intraclass correlation coefficients will be interpreted as poor agreement (<0.40), weak agreement (0.40–0.54), moderate agreement (0.55–0.69), good agreement (0.70–0.84) or excellent agreement (0.85–1.00) [30].

### 2.7 Power calculation

We carried out a power calculation for the paired *t*-test investigating the participants’ change in DERS-T at from baseline to end-of-treatment (Table 1, *model 1a*). Based on a power of 80%, an alpha of 0.05, a standard deviation on DERS-T at baseline of 21.72 [31], and number of participants in the smallest group of 57, we will be able to detect a mean difference on DERS-T from baseline to end-of-treatment of 11.51 points.

### 2.8 General analysis principles

Statistical analyses will be performed using the latest available stable version of R (R Core Team, Vienna, Austria) and SPSS version 28 (IBM Corp. Released 2021. IBM SPSS Statistics for Windows, Version 28.0. Armonk, NY: IBM Corp.). We will use at least two different softwares for each analysis to minimize type-1 error caused by software specific differences [32]. If the level of significance and/or effect sizes differ between the softwares, the most conservative result will be reported. For the mediation analysis in SPSS, we will use the PROCESS Macro level 4 [33]. The analyses will be intention-to-treat analyses, but ultimately carried out as complete case analyses (see *Handling of missing data*). Missingness, dropouts, and discontinuation of treatment will be reported. For the pooled analysis of participants, CY-BOCS will be treated as a continuous predictor variable (Table 1*; models 1a, 2a, 3a, 4a*). For the analysis of allocation, CY-BOCS will be treated as a dichotomous predictor variable (16–23 compared to ≥ 24 points) (Table 1*; models 1b, 2b, 3b, 4b*), mimicking the stratification of the trial [19].

### 2.9 Handling of missing data

Overall, no imputations will be carried out, and analyses will be carried out using all available data. Specifically, for DERS-T, if more than 25% of items on any subscale is missing for individual responses, the questionnaire including the DERS-T will be removed from analyses. For missing items below 25% per subscale, prorating procedures will be used by taking the mean of the available scores from the relevant subscale and multiplying them by the number of items on that subscale.

### 2.10 Assessments of underlying statistical assumptions

We will assess the statistical assumptions underlying each statistical model. All variables will be visually inspected and assessed for normal distribution using histograms and quantile-quantile plots. For all regression models we will use bootstrap methods around the mean and 95% confidence interval if distributions do not allow for conventional variance defined confidence intervals. We will not adjust for multiple comparisons since the study is exploratory. However, we will report estimands, confidence intervals, and effect sizes for all tests possible and consider the risk of false discoveries in the interpretation of our results.

## 3 Discussion

Here, we outline a combined protocol and statistical analysis plan for an exploratory study from the TECTO trial focusing on the relationship between changes in emotion dysregulation and the association with psychotherapy effects for youths with OCD.

The blended study design includes three participant groups: an experimental group receiving FCBT, an active control group receiving FPRT, and a healthy control group. Participants in both intervention groups were offered 14 sessions of manualized treatment with the main and intended difference being the absence of the ERP component in the FRPT arm of the trial [17]. The data from the healthy control group allow us to assess if the proportion of participants with OCD reporting normative levels of emotion regulation changes from before to after psychotherapy. Importantly, no waitlist group of participants with OCD is included in the study, which may limit our ability to definitively link psychotherapy to improvements in emotion regulation and vice versa rather than the mere passage of time. Although the healthy control group provides a normative baseline and thus a proxy for the developmental changes of emotion regulation over time, it does not account for the potential naturally occurring changes in emotion regulation abilities in youths with OCD during the study period. Notably though, if future studies include waitlist groups to enhance robustness and interpretability of our findings, it is important to stress that these designs may also present their own challenges [34,35].

All outcomes in this statistical analysis plan are assessed in a two-fold manner. First, we will combine the FCBT and FPRT groups to assess the interplay between emotion dysregulation and psychotherapy. These combined models are based on work suggesting that common elements, e.g., setting treatment goals and learning acceptance, across different types of interventions may be effective in targeting problems with emotion regulation and mental health in youths [36,37]. Second, we compare the treatment groups by allocation to more directly assess the ERP component proposed as the active mechanism of change for OCD [17]. To date, a few studies have examined the interplay between emotion dysregulation and CBT with EPR in youths with OCD [7,8,10]. However, to our knowledge no studies have employed a design to try to isolate the ERP component as the active ingredient.

### 3.1 Strengths

The primary strengths of this predefined statistical analysis plan are 1) to ensure transparency by detailing the methods and procedures to be employed in the result paper and 2) to enhance reproducibility by preventing data-driven decisions and selective reporting.

### 3.2 Limitations

The primary limitation is the risk of false discoveries given our relatively small sample size, albeit larger than most other emotion regulation studies on pediatric OCD. Therefore, the result of any model except for *model 1a* should be interpreted with caution and at best be hypothesis generating for future studies. Second, results from the baseline assessments of emotion dysregulation (including subjective, behavioral, and psychophysiological indices) have been submitted for publication [31]. This work has guided the choice of outcome measures for the longitudinal study, and our decision could thus be argued to be biased by our preliminary findings. Third, youth age was used as a stratification variable in the trial (8–12 compared to 13–17 years) [19]. However, given that the DERS was only completed by children aged 11 years or above we are not able to use this stratification variable in our analyses.

## 4 Conclusion

This statistical analysis plan for the exploratory emotion regulation study includes a detailed predefined description of how data will be analysed. We have included descriptions of the statistical considerations. Statistical analysis plans limit selective reporting bias. This statistical analysis plan will increase the validity of the results of our blended study.

## CRediT authorship contribution statement

**Christine Lykke Thoustrup:** Writing – original draft, Project administration, Methodology, Investigation, Funding acquisition, Conceptualization. **Camilla Uhre:** Writing – review & editing, Investigation. **Valdemar Uhre:** Writing – review & editing, Investigation. **Melanie Ritter:** Writing – review & editing, Investigation. **Signe Vangkilde:** Writing – review & editing, Methodology. **Janus Engstrøm:** Writing – review & editing, Methodology. **Jane Lindschou:** Writing – review & editing, Methodology. **Christian Gluud:** Writing – review & editing, Methodology. **Anne Katrine Pagsberg:** Writing – review & editing, Project administration, Conceptualization. **Markus Harboe Olsen:** Writing – original draft, Validation, Supervision, Project administration, Methodology, Investigation, Conceptualization.

## Ethics approval and consent to participate

The Ethics Committee of the Capital Region of Denmark, approval number: H-18010607, and the Knowledge Centre on Data Protection Compliance in the Capital Region of Denmark: VD-2018-263, I-Suite no.: 6502.

## Data availability

No data were used in the current study. Data used in the planned study will be available in a deidentified dataset on the Zenodo database after results from the study and the TECTO trial have been published.

## Funding

This work was supported by the Capital Region 10.13039/100020584Mental Health Services Research Fund. The funding source was not involved in the study design, data collection, or analysis plan.

## Declaration of competing interest

The authors declare the following financial interests/personal relationships which may be considered as potential competing interests: Valdemar Funch Uhre is now employed by Novo Nordisk A/S.
